# Testing macroecological theories in cryptocurrency market: neutral models cannot describe diversity patterns and their variation

**DOI:** 10.1098/rsos.212005

**Published:** 2022-04-13

**Authors:** Edgardo Brigatti, Estevan Augusto Amazonas Mendes

**Affiliations:** Instituto de Física, Universidade Federal do Rio de Janeiro, Av. Athos da Silveira Ramos 149, Cidade Universitária, 21941-972 Rio de Janeiro, Brazil

**Keywords:** cryptocurrency market, macroecological theories, neutral model

## Abstract

We develop an analysis of the cryptocurrency market borrowing methods and concepts from ecology. This approach makes it possible to identify specific diversity patterns and their variation, in close analogy with ecological systems, and to characterize the cryptocurrency market in an effective way. At the same time, it shows how non-biological systems can have an important role in contrasting different ecological theories and in testing the use of neutral models. The study of the cryptocurrencies abundance distribution and the evolution of the community structure strongly indicates that these statistical patterns are not consistent with neutrality. In particular, the necessity to increase the temporal change in community composition when the number of cryptocurrencies grows, suggests that their interactions are not necessarily weak. The analysis of the intraspecific and interspecific interdependency supports this fact and demonstrates the presence of a market sector influenced by mutualistic relations. These latest findings challenge the hypothesis of weakly interacting symmetric species, the postulate at the heart of neutral models.

## 1. Introduction

Since the appearance of Bitcoin, the first peer-to-peer digital currency, introduced by a paper authored under the pseudonym of Satoshi Nakamoto in 2008 [1], the cryptocurrency market has known a spectacular growth in terms of capitalization and the number of different cryptocurrencies. This success arouses economists’ interest, engaged in exploring if these digital currencies could perform all the three functions of money (medium of exchange, unit of account and store of value) [2] and worried about the effects that this trend could produce on traditional monetary or governmental authorities.

As most cryptocurrencies are used more like a portfolio speculative asset than as a currency, they attracted the attention of traders and institutional investors. This trend has been reflected in academic studies, that generally have focused on Bitcoin and its price dynamics [3–5]. More recently, some studies have tried to describe the overall market [6], looking at the dynamics of all the cryptocurrencies actively traded [7,8]. Among these last works, ElBahrawy *et al.* [7] introduced an interesting perspective looking at the cryptocurrencies market as an ecological system. Based on this frame of mind, they described several typical distributions. The use of this parallelism is not new and follows a stream of works where non-biological [9,10], synthetic systems [11–13] or artificial-life type simulations [14] have been described in terms of ecological systems and used for testing ecological and evolutive theories. In this work, we will focus on these aspects, developing an in-depth analysis of the cryptocurrencies market either for describing some of its fundamental aspects or for comparing and evaluating ecological models.

In the last two decades, two contrasting ecological theories generated an important debate around the principal mechanisms that shape ecological communities. Niche theories [15,16] state that the most relevant factors that determine communities are defined by the selection produced by the interactions among the individuals, the species and the environment. By contrast, neutral theories [17,18] consider that the dominant factors are the stochastic processes present in populations dynamics, which determine their random drift.

In its more successful implementation, the neutral theory of biodiversity models the organisms of a community with identical *per capita* birth, death, immigration and speciation rates [17]. There are no differences among the species, which are considered demographically and ecologically identical. These hypotheses imply, from a theoretical perspective, the assumption of functional equivalence: on the same trophic level, species are characterized by identical rates of vital events. From a model perspective, they lead to the symmetry postulate: the dynamics of the community is not influenced by interchanging the species labels of individuals [19].

In this work, we will consider only non-spatial neutral models which describe populations at the level of individuals, not of species, allowing the direct estimation of species abundances. Among these models, we consider very general Markovian models, usually described through a master equation [18] or a Langevin equation [20]. These approaches generate predictions at stationarity. For this reason, we will focus our attention on variables that can be considered close to stationarity.

These ecologically neutral models generate statistically neutral distributions of different macroecological patterns. In general, to test these theories with empirical data, it is determined, using a statistical selection, if such distributions are compatible with the empirical ones. Strictly speaking, this statistical neutrality (the adherence between the model and data distributions) is a necessary but not sufficient condition for claiming neutrality [21]. Even if this is the most pragmatic way for testing neutrality, the analysis of the form of interactions and if species satisfy the assumption of functional equivalence could produce more enlightening and definitive results.

Based on these considerations, the principal aim of our study will be the analysis and characterization of different macroecological patterns converted to the specific case of the cryptocurrency market. Furthermore, we will focus our attention on the examination of the interdependence and correlations present among cryptocurrencies. In the section on Methods, we will present in detail which patterns and how these analyses can be carried out.

In brief, the construction of an analogy between the cryptocurrency market and an ecological system will better elucidate the community structure of the cryptocurrency market, shedding light on the existing relationships between cryptocurrencies. Despite its importance, the theoretical description of structure and interactions in markets is limited, and new concepts and approaches are required. Ecology is capable of introducing new and powerful ideas, previously debated and tested. In this work, we will describe specific dynamics, determine new stylized facts and patterns and compare them with a testable theory. This analysis will bring new insights and comprehension of the considered market, highlighting important elements which could be useful for assisting in hedging risks. The natural limitation of an analogy-based approach could be overcome in the future by introducing specific features for correcting inaccuracies and building new models.

## 2. Data

We collected the cryptocurrencies data from the website Coin Market Cap [22], which extracts from the exchange market platforms the price expressed in US dollars (exchange rate), the volume of trading in the preceding 24 h and the market capitalization of the different cryptocurrencies. Market capitalization is the product of the price for the circulating supply, which cannot account for dormant or destroyed coins. Traded cryptocurrencies can disappear from the website list to reappear later on. In fact, capitalization of cryptocurrencies not traded in the 6 h preceding the weekly report is not included in the dataset and cryptocurrencies inactive for 7 days are not included in the list. For this reason, we filled these lacunae by introducing the average between the values available at the extremes of each gap. Finally, we cleared the dataset by correcting some mismatches or typos present in the names or symbols of the cryptocurrencies.

We considered weekly data from 28 April 2013 to 2 February 2020, which correspond to a series of 354-time steps. The dataset contains a total of 3588 cryptocurrencies.

## 3. Methods

To construct the parallelism between ecological systems and the cryptocurrency market, we consider that each cryptocurrency represents a species, and its capitalization its abundance. In this way, the wealth invested in a cryptocurrency replaces the population size of a species.

The first macroecological pattern that we analyse is the species abundance distribution (SAD), a classical biodiversity descriptor that characterizes static features of ecosystem diversity. This distribution represents the probability that a species presents a given population size. By using a very general stochastic Markovian model for neutral ecological communities, Volkov *et al.* showed that, at stationarity, the SAD follows an analytic zero-sum multinomial distribution for local communities and, for the metacommunity, the celebrated Fisher’s distribution [18,23], as already predicted by Hubbell’s neutral model [17]. Note that, as the model is non-spatial, the term metacommunity represents a single, permanent large community where migration is absent. By contrast, local communities present dynamics defined by immigration from a permanent source of species (the metacommunity). In this sense, our data must be considered as collected from the metacommunity and, based on the results of Volkov *et al.,* they should be described by Fisher’s distribution: *p*(*x*) ∝ e^−*cx*^/*x* [24]. A classical alternative to the distribution produced by the neutral theory is the lognormal one: $p(x)=(1/\sqrt{2\pi \sigma^{2}}x)\;e^{-{\left(\log x-\mu \right)}^{2}/2\sigma^{2}}$, which is equivalent to a normal distribution if the variable log(*x*) is chosen. This distribution has a long tradition of use in the ecological literature [25], and it is a reasonable and parsimonious null hypothesis for the SAD [26], which can be obtained from pure statistical non-biological arguments, based on the central limit theorem [27], or can be generated by niche or demographic differences among species in population models [28]. To sum up, a description of the SAD with Fisher’s distribution would support the idea of a neutral dynamics. By contrast, a lognormal distribution would suggest the presence of other types of population dynamics or pure statistical mechanisms.

The second macroecological pattern is the species population relation (SPR). It describes the scaling of the number of species *N* with the total number of individuals *x*. When the different species of a total population *x* follow Fisher’s distribution, the expected number of species for a given population *x* is given by: *N*(*x*) = *α*log(1 + (*x*/*α*)) [24]. Ecological studies usually measure the relationship between the number of species and the area sampled, which can be easily obtained assuming a linear relation between *x* and the area. An alternative characterization of this relation commonly found in the literature is based on empirical curves showing a power-law behaviour, with exponents presenting typical values between 0 and 1 [29].

So far, we have focused on static macroecological patterns which can be related to results generated by neutral theories at the steady state. However, stationarity is just an approximation, and not necessarily a good one, for systems characterized by a state of flux in species, abundance and composition. For this reason, we look at the temporal behaviour of our system and characterize time-dependent patterns.

The intertwined history of different species along their evolution generates complex communities characterized by non-trivial structures. New species continuously replace old ones producing an intricate overlap where the turnover can be characterized employing the species turnover distribution (STD). This distribution is defined as the probability that the ratio of the population of a species separated by a time interval *t*, *x*(*t*)/*x*(0), is equal to *λ*. Under stationary conditions, Azaele *et al.* [20] introduced a neutral model that can forecast the STD. Within this framework, an analytic expression for this distribution is obtained

3.1 $$P_{\text{STD}}(\lambda ,t)=C\frac{\lambda +1}{\lambda }\frac{{\left({\text{e}}^{t/B}\right)}^{A/2}}{1-{\text{e}}^{-t/B}}{\left[\frac{\sinh(t/2B)}{\lambda }\right]}^{A+1}{\left[\frac{4\lambda^{2}}{{\left(\lambda +1\right)}^{2}{\text{e}}^{t/B}-4\lambda }\right]}^{A+1/2},$$

where *A* and *B* are parameters and *C* is a renormalization constant equal to $\frac{2^{A-1}}{\sqrt{\pi }}\frac{\Gamma (A+1/2)}{\Gamma (A)}$.

The community composition of cryptocurrencies presents a coherent structure that evolves over time. This structure can be quantified by analysing how the capitalization of each coin changes. A variety of different indices has been used in ecology for tracking modifications in community composition through a similarity measure [30,31]. Here, we adopt a simple and well-known one that presents a straightforward interpretation of its values [32]. Given the capitalization *C*_*i*_(*t*) of a species *i*, we consider $S_{i}^{t}=\log(C_{i}(t)+1)$ (1 is added in order to avoid the log of 0), where *t* is a given month. Note that the log-transformation is a natural approach for quantities which can be roughly described by a lognormal distribution. The next step is the estimation of Pearson’s correlation of the log-transformed data: $r_{S}(\tau )=\mathrm{Corr}(S_{i}^{t_{0}},S_{i}^{t_{0}+\tau })$, where the correlation is calculated over the index *i*. This method is well known [32,33] and easily supports tests of significance: 0 represents complete randomness and is the null hypothesis for significance tests. To conclude, we characterize the mode of the community similarity evolution by looking at the functional form of the temporal decay of *r*_*S*_, which describes the transformation from perfectly correlated structures towards totally uncorrelated ones.

We compare the results obtained from this empirical analysis of the community composition with the ones generated by a neutral model introduced in [20,34], which is constituted by a system of stochastic discrete differential equations. The model describes an ecological community with a fixed number *s* of species, where the total number of individuals in the community is set to *N*. In the large population limit, these constraints can be relaxed and similar results are obtained [34]. If $x_{i}^{t}$ represents the population of the *i*th species at time *t*, its evolution follows this equation

3.2 $$x_{i}^{t+1}=N\frac{\rho x_{i}^{t}+\sigma \sqrt{x_{i}^{t}}\eta_{i}^{t}+b}{\sum_{\;j=1}^{s}(\rho x_{j}^{t}+\sigma \sqrt{x_{j}^{t}}\eta_{j}^{t}+b)}.$$

The parameter *b* controls the population size near the extinction threshold, taking into account the total effect of immigrations, extinctions and speciations. *ρ* and *σ* are the mean value and the standard deviation of the distribution which describes the *per capita* birth rate. Based on the principle of neutrality, we assume that *ρ* and *σ* are the same for all individuals. Finally, $\eta_{i}^{t}$ is an uncorrelated Gaussian noise term, with zero mean and unit variance. By taking the continuum limit, this stochastic discrete model can be approximated by a Langevin and the associated Fokker–Plank equation. Its analytical solution shows that, in the limit of large *N*, a Gamma distribution describes the SAD for this model. Such distribution can well describe various experimental SAD data and approaches Fisher’s distribution for 2*b*/*σ*^2^ <<1 [20,34]. These results can be connected with the classical outcomes of the neutral theory as formulated by Volkov *et al.* [18] for a particular choice of the birth and death coefficients.

Our last analysis looks at the characterization of the interdependence among cryptocurrencies. This inspection will help in exploring if in a community of cryptocurrencies the symmetric species postulate is well supported and if interactions between species can be considered weak in relation to stochastic effects, as expected for neutral dynamics. As capitalizations present a growing trend, we do not study directly their correlations. By contrast, we consider the log-variation of the capitalization of a given species *i*: *V*_*i*_(*t*) = ln[*C*_*i*_(*t*)] − ln[*C*_*i*_(*t* − 1)]. As a dependence measure, we consider Pearson’s correlation between the synchronous time evolution of *V*(*t*) of a pair of cryptocurrencies *i* and *j*: $r_{V}^{ij}=\mathrm{Corr}(V_{i}(t),V_{j}(t))$. The interdependencies among the log-variations in the capitalization are addressed looking at the corresponding correlation matrix.

## 4. Results

### 4.1. General dynamics

**Figure 1.**
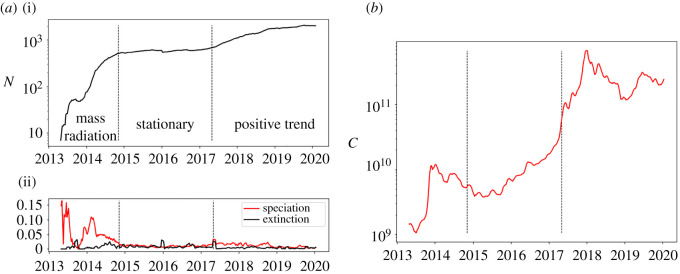
(*a*) Number of actively traded cryptocurrencies (i), speciation and extinction rates during a given week averaged over rolling windows of four weeks (ii). The dashed vertical lines represent the starting of the stationary phase (2 November 2014) and its ending (30 April 2017). (*b*) Market capitalization averaged over rolling windows of four weeks.

We consider the time series which collect the number of active cryptocurrencies at a given time (*N*(*t*)) and we evaluate the corresponding speciation and extinction rates (figure 1), measured as the number of cryptocurrencies entering (respectively, leaving) the market on a given week, normalized over the number of active cryptocurrencies at that time. By looking at the number of active cryptocurrencies and at the speciation and extinction rates, it is possible to qualitatively discriminate between three different regimes: before 1 June 2014 a mass radiation phase, characterized by a spectacular increase in the number of cryptocurrencies, caused by a very high number of speciation events. This phase is characterized by significant fluctuations. Between 2 November 2014 and 30 April 2017, we can highlight a stationary phase, with comparable values of speciation and extinction rates. Starting from 7 May 2017, a positive trend characterizes a new regime where the number of cryptocurrencies grows slightly in a gradual and regular fashion. Also, this regime presents higher values of speciation rates in relation to the extinction ones. In general, extinction rates fluctuate around a typical value, except for some sparse and sudden extinction bursts. By contrast, speciation rates present different behaviours which determine the modes of the three considered regimes.

The total market capitalization *C*(*t*) in general presents important positive trends. In the regime where the number of active cryptocurrencies is stationary, intervals of exponential growth can be detected; in the regime with important radiation of new currencies, stages with super-exponential behaviours can be identified.

### 4.2. Species abundance distribution

**Figure 2.**
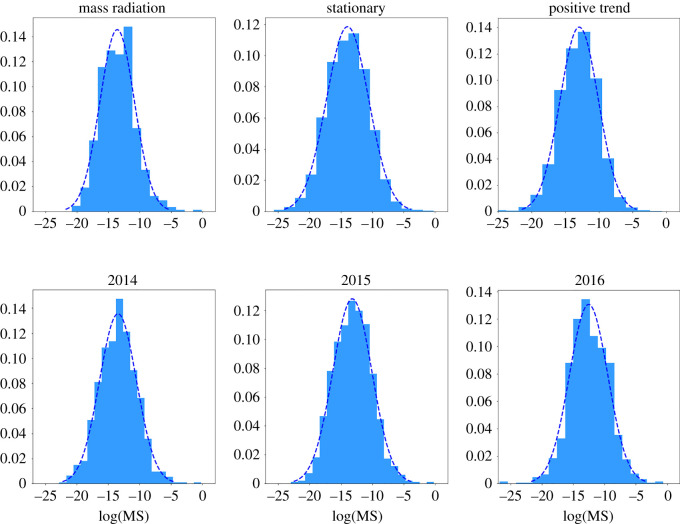
Species abundance distribution sampling the empirical data along different periods. Top: data sampled during the periods of mass radiation, the stationary period and the positive trend. The *p*-values of the Kolmogorov–Smirnov test are 0.584, 0.626, 0.245 for datasets presenting 652, 1291 and 2825 elements. Bottom: data collected during the stationary period, starting in November 2011, and sampling along 1 year. The *p*-values of the Kolmogorov–Smirnov test are 0.996, 0.715, 0.253 for datasets presenting 840, 866 and 1170 elements.

Figure 2 shows the SADs considering different periods and time scales. We present data aggregated along a year or more, considering all the stationary and non-stationary periods. For each cryptocurrency, we measure the corresponding market share (MS) for overcoming the problem of the non-stationarity of the capitalization. Comparable results can be obtained for shorter time scales, corresponding to a month or even a week. For testing the neutral theory, we must focus on the stationary period, where we can consider the dynamics in the number of active cryptocurrencies as stationary and we can compare the empirical data to the theoretical predictions, which are obtained at stationarity. It is important to note that, in ecology, the situation of incompletely censused regions is frequent. This condition usually produces an under-sampling of rare species. In this situation, it is common to consider to fit only the portion of the distribution which encompasses the most common species. By contrast, in our case, we can consider having access to a fully sampled community, an assumption supported by the regularity of the shape of the left tail of the distribution, which does not suffer from strong statistical fluctuations. Just by looking at the full shape of our distributions, which always present an evident internal mode, we can firmly discard the hypothesis that Fisher’s distribution can give a good description of our dataset. In fact, Fisher’s distribution is monotonic and does not present internal modes. By contrast, we can perform a high-quality fitting using the lognormal distribution. In particular, by using a Kolmogorov–Smirnov test of our observations against the fitted lognormal distributions, we cannot reject the hypothesis that the data come from the fitted distribution since *p*-values are very high in all the considered cases (figure 2).

### 4.3. Species population relation

**Figure 3.**
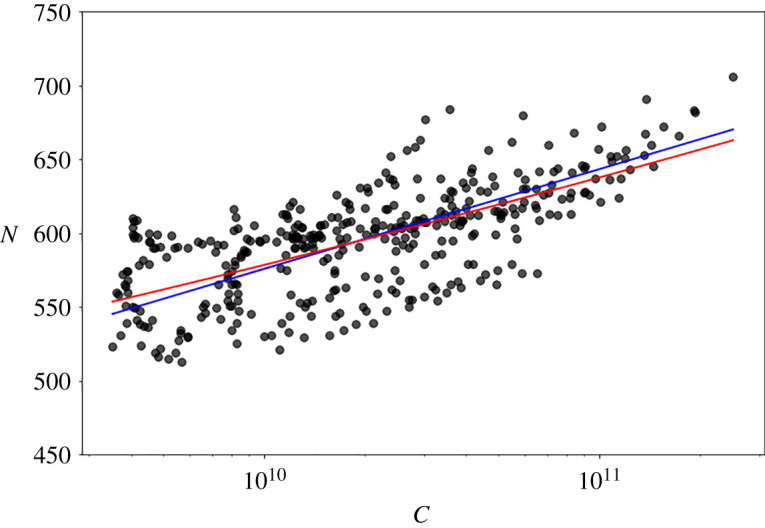
Log-linear plot of the capitalization versus the number of species. The red line is the best fitting obtained from the relation: *α*log(1 + (*x*/*α*)), the blue one from a power law.

For studying the SPR, we analyse the stationary period and scan the corresponding dataset with different temporal intervals. We consider intervals between 1 and 10 weeks and, for each sample, we measure the value of capitalization and number of species. We plot the pairs of all these values in figure 3, where we represent the fitting obtained by using the relation *α*log(1 + (*x*/*α*)) compared with a power law. The dependence of *N* on *C* is very weak and it is not possible to affirm if the logarithmic or the power-law function better describes the data points.

### 4.4. Evolution of the community structure

**Figure 4.**
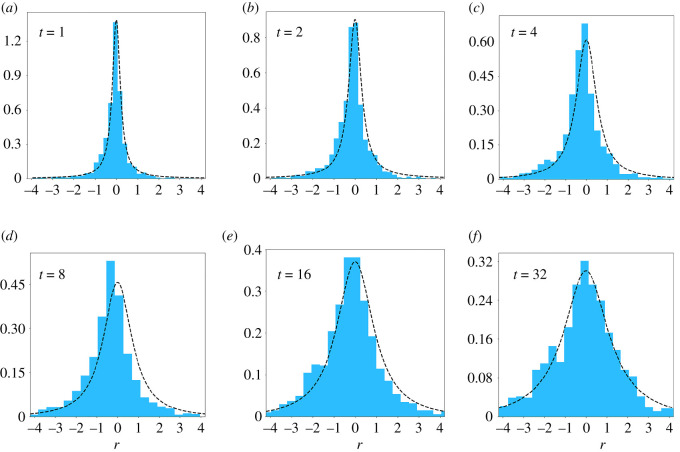
Species turnover distribution evaluated considering the cryptocurrencies market share. We fix *t* = 1, 2, 4, 8, 16, 32 weeks. We consider the variable *r* = log(*λ*) and plot the distribution *P*′(*r*) = e^*r*^*P*_STD_(e^*r*^, *t*), where *P*_STD_(e^*r*^, *t*) is given by equation (3.1). The best fittings are represented by the dashed lines.

We estimate the STD using the cryptocurrencies market share. We consider coins with *x*(0) located in the stationary period. The fittings of the STD with the analytic expression of equation (3.1) present contrasting results, as can be appreciated in figure 4. For some values of *t*, the fitting is satisfactory, for others, there is a small but systematic difference between fitted and empirical distributions. Empirical data present an asymmetry between left and right tails, with a greater propensity of developing negative *λ* values, which correspond to decreasing populations. The parameters obtained from the fitting of the STD can be used to produce the SAD generated by the neutral model of [20,34]. The fitted values of *B* (0.48 ≤ *B* ≤ 0.71) lead to SADs presenting a shape far from the expected for neutral models describing a single community without migration.

**Figure 5.**
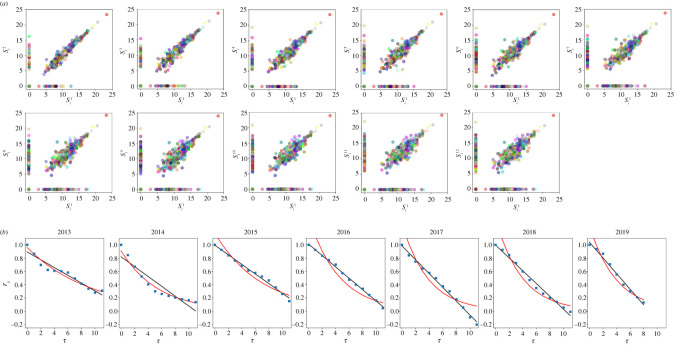
(*a*) Scattering plot of the log-abundances in the initial community versus abundances at the following specified time, expressed in months. Data are displayed for the interval from 28 April 2015 to 28 April 2016. As a result, initially rare or absent species appear near the *x*-axis and rare or extinct species appear near the *y*-axis. Note how numerous cryptocurrencies go extinct. Different colours correspond to different currencies. (*b*) The evolution of the community structure index *r*_*S*_ during 1 year. Data starts from 28 April 2013. The continuous black lines are the best fitted linear functions; red lines represent the exponential fittings. Note that, in general, correlation presents positive values. Anyway, negative values can be reached, as a product of the final accumulation of species around the *x*- and *y*-axis.

**Figure 6.**
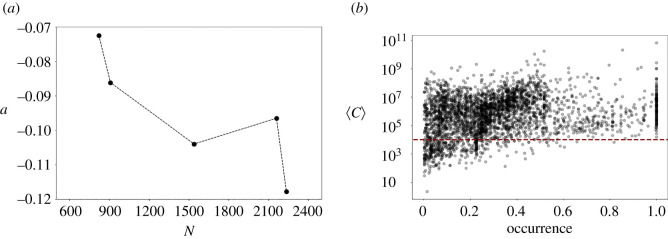
(*a*) The regression slopes of the best fitted linear functions (*a*) versus the number of active cryptocurrencies (species richness) for the five temporal intervals considered in figure 5 from 2015 to 2019. The first quantity is a measure of temporal *β*-diversity (species turnover, as the variation in species composition through time), the second of *α*-diversity. (*b*) Scattering plot of the occurrence versus the mean capitalization of a given cryptocurrency. Data are evaluated considering the interval from the beginning of the stationary period to the end of the dataset.

Figure 5 shows an example of the scattering plot of the log-abundances in the initial community versus abundances at the following specified time. For these different plots, we evaluated the correlations and we examined their evolution along 1 year. We perform this analysis for all our data. In the period of mass radiation, we can observe an exponential behaviour. By contrast, as the community enters the stationary regime, the *r*_*S*_ decreases with time following a clear linear behaviour and this mode is maintained until the end of our time series. The slope values of the linear fittings (*a*) decrease along the considered years. These slope values are an interesting parameter for quantifying what ecologists call temporal *β*-diversity [31], which characterizes the change in the composition of a single community through time. More negative *a* values represent a more intense variation in the community structure, which corresponds to a stronger temporal *β*-diversity. In this framework, the relation between temporal *α*-diversity, which characterizes the temporal variation of species in a single community, and temporal *β*-diversity is particularly interesting. In figure 6, we can appreciate a general trend of increasing temporal *β*-diversity with increasing *α*-diversity. *α*-diversity is estimated using a simple count of the number of species (species richness) in the considered interval.

In addition, we analyse if a clear relationship exists between the abundance of the population of a given species and its lifetime. We estimated the abundance considering the mean value of the capitalization of a species estimated over its time series. The lifetime is obtained by measuring the occurrence of the fraction of weeks in which a cryptocurrency is active over all the considered weeks. By looking at the scattering plot of these quantities (figure 6), we can see that cryptocurrencies with a capitalization smaller than 10^4^ present a reduced lifespan, with occurrences that hardly reach 0.5. However, we can perceive that these quantities display an unexpected rather weak dependence.

We conclude this analysis by comparing the *r*_*S*_ behaviour displayed during the stationary period with the outcome of the neutral model presented in equation (3.2). Simulations are run for a number of generations that allows reaching *r*_*S*_ values close to the smallest one displayed by real data. We fix the number of species *s* considering the mean number of species appearing in the dataset during the chosen periods, and we select the ratio 2*b*/*σ*^2^ among values that are small enough for generating distributions close to Fisher’s one. This purpose is reached by fixing *b* = 1 and varying *σ*.

**Figure 7.**
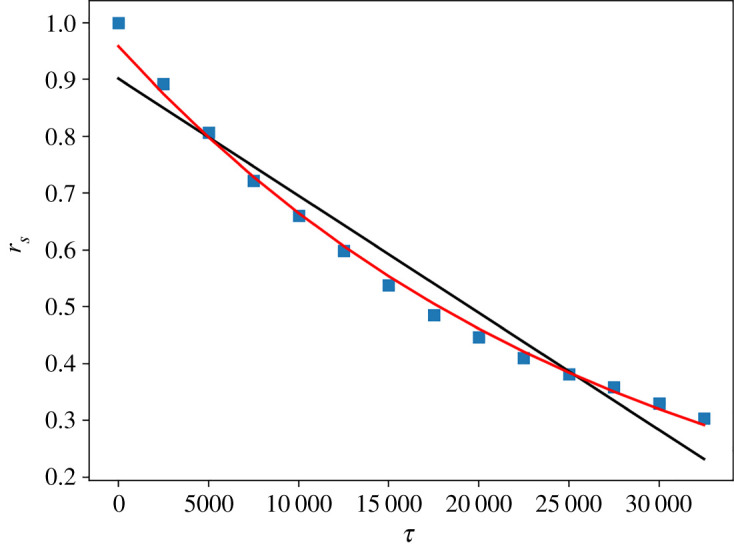
A typical *r*_*S*_ decay obtained from the artificial data generated by the neutral model of equation (3.2). In this case, the model parameters are: *N* = 10^7^, *ρ* = 3.5, $\sigma =\sqrt{20}$, *b* = 1 and *s* = 1500; *τ* are expressed in simulation times. The continuous black line is the linear fitting, the red line the exponential one.

We collect the species abundances and take into account the extinction/speciation events. These events are calculated when the term $\rho x_{i}^{t}+\sigma \sqrt{x_{i}^{t}}\eta_{i}^{t}$ crosses a zero value. From these artificial data, we can obtain the *r*_*S*_ values. We run a large amount of simulations for different values of *N*, *ρ* and for $\sigma >\sqrt{3}$. For this range of parameters values, the model generates only exponential decays. Figure 7 shows a typical example for specific parameters.

### 4.5. Correlations

**Figure 8.**
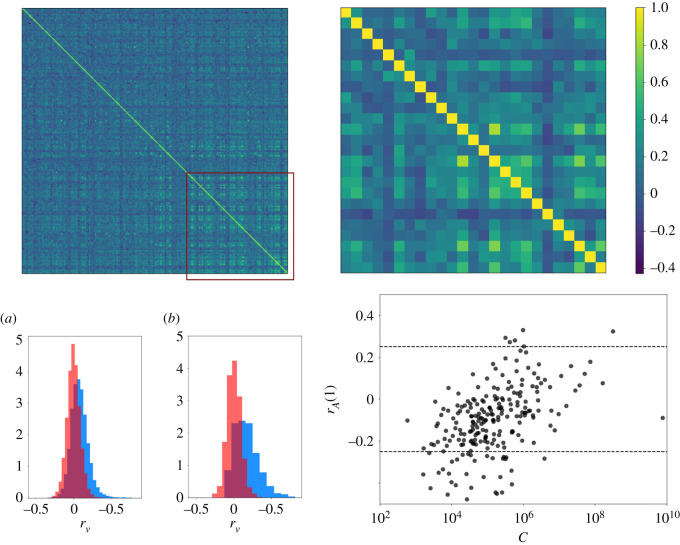
Top: plots of correlation matrices for all the considered cryptocurrencies (left) and for the top 25 (right). Cryptocurrencies fill the matrix from the smallest to the largest capitalized ones (the considered capitalization is the total capitalization over the stationary period). Note how the pairs of highly capitalized cryptocurrencies (highlighted by the red square) present, in general, higher correlations. Bottom: On the left, in blue, are the distributions of the pair correlations for all the considered cryptocurrencies (*a*) and for the top 25 (*b*). In red, the same distributions obtained from the random shuffled series. On the right, the temporal autocorrelation for *τ* = 1 as a function of the aggregated capitalization over the stationary period of the cryptocurrencies. The dashed lines represent the 1/99 percentiles of the distribution of the autocorrelations of the randomized data estimated over 10 different realizations.

We studied the subset of 225 cryptocurrencies that are active during the entire stationary period. The correlation matrix for pairs of cryptocurrencies is reported in figure 8. A qualitative observation of the correlation values shows a clear deviation from the values of the randomized sample and the matrix presents a clear structure. For the cryptocurrencies with higher capitalization, correlations have larger values, which are statistically significant and positive. This result can be clearly appreciated by looking at the behaviour of the largest 25 cryptocurrencies.

Finally, we look at the temporal autocorrelation for a single cryptocurrency: *r*_*A*_(*τ*) = Corr(*V*(*t*), *V*(*t* + *τ*)). We focus on *r*_*A*_(1), as for *τ* > 1 autocorrelation values are in general indistinguishable from the ones of a random series. As shown in figure 8, there is a clear dependence of *r*_*A*_(1) on the capitalization of the considered cryptocurrencies. For smaller capitalizations, some cryptocurrencies present a significant anticorrelation, for larger capitalizations only a few cryptocurrencies show a significant, but small, positive correlation. In particular, among the largest 25 currencies only two present significant correlation values.

## 5. Discussion

The shape of the SADs lends strong support to alternatives to the neutral theory. In fact, neutral models without migration, which describe the distribution at the metacommunity scale, predict a Fisher’s distribution. By contrast, tests of fit quality show that the lognormal approximates the observed species abundance data very well. This means that looking at the log-scale, the SAD shows a strong central tendency and relatively few rare or abundant species. By contrast, neutral dynamics would generate more rare species and truly dominant ones would be improbable.

The analysis of the SPR gives no definitive answers that would allow the selection of one of the two alternative models. In fact, a dependence between species and population exists, but it is very weak and it is not possible to distinguish between a logarithmic or a power-law dependence.

The STD fitting with the distribution generated by a neutral model does not allow a straightforward interpretation. Even if the fitting, for some values of *t*, is promising, we must stress that the same distribution fits equally well turnover data generated by very different community populations, corresponding to distinct SADs, which can describe situations with or without central modes. For this reason, it is important to look at what SAD is produced by a given STD fitting. In our case, the STD fittings generate SADs presenting a shape far from the expected for neutral models describing a single community without migration. For this reason, we think that this result is not supporting the neutral theory. This specific example shows how curve-fitting is a valuable approach to test a model, but it is not necessarily conclusive. In particular, this is the case when the fitting distributions are flexible enough for describing very different situations.

The evolution of the community structure correlations shows a very interesting mode. In fact, after the radiation phase, it displays a characteristic linear decay. The considered neutral model describing a single community without migration cannot generate this linear behaviour. The absence of an exponential behaviour suggests that the dynamics of the change in the community structure is very far from being random, with a quite slow drift in the relative abundances of moderately abundant species. The system is apparently endowed with a mechanism, not present in the neutral model, which allows relatively rare and moderately abundant coins to persist over time. This conjecture is supported by the performance of the cryptocurrencies persistence in relation to their abundance: only very rare coins effectively go systematically extinct. Persistence and abundance display a weak dependence and, in contrast with the results of neutral models (see [20]), it is not evident that the less abundant species are clearly more prone to extinction.

The relation of the pace of change in the community structure with its species richness shows how the presence of more cryptocurrencies tends to accelerate the reorganization of the composition of the community over time. If a considerable amount of new species enter the system, the market must accommodate via a faster temporal reorganization which can generate a finer temporal subdivision of the wealth injected in the market. The fact that an increase in *α*-diversity has a direct effect on temporal *β*-diversity, may suggest that interspecific competition among cryptocurrencies is not so weak.

These original results demonstrate the particular advantage of using non-biological data for testing and improving ecological theories and methodologies in the quantification of temporal behaviours, where ecological datasets are generally scarce or must rely on fragmented palaeontological data.

The analysis of the correlation matrix for pairs of cryptocurrencies quantifies the dependence of the increase/decrease of a species *i* on the increase/decrease of a species *j*. Results clearly show the presence of a group of coins, included among the higher capitalized cryptocurrencies, presenting a cohesive response in the variation of their capitalization. This important result shows a coherent behaviour in a sector of the market. The positive and statistically relevant correlations suggest the presence of mutualism: cryptocurrencies that belong to this sector benefit from an increase in the capitalization of the other cryptocurrencies which belong to the same sector. This effect can be determined either by endogenous factors or by the eventual contribution of exogenous common cause drivers. We can distinguish between two classes of cryptocurrencies: a minor one where the variation of the capitalization presents clear positive correlations, and a larger one, where this variation is uncorrelated. At the considered time scale, there is no switch between these classes and we can label species as belonging or not to a given class. Cryptocurrencies are not symmetric in relation to this behaviour and the symmetric species postulate seems not to be satisfied, implying that the system is not neutral. In neutral models, common species are treated simply as rare species, but here the behaviour of the correlations suggests that different mechanisms are shaping these two classes of cryptocurrencies and should be taken into account. Coins with larger capitalization cannot be treated simply as rare coins writ large [35]. We remember that, in ecologically neutral models, the correlation in the abundances of a pair of species is the same as the correlation in the abundances of any other pair of species [21]. Thus, a distribution with equal correlation coefficients can stand in as a proxy for measuring statistical neutrality. A similar role can be assumed by the correlations between pairs of *V*_*i*_(*t*).

Finally, we can shed a light on the relevance of the interactions between species, and if they are effectively weak compared with the stochastic drivers of the dynamics, by contrasting the values of the correlation among pairs of cryptocurrencies with the autocorrelation of each cryptocurrency. The pair correlations can be seen as a proxy for between-species interdependency (interspecific) and the autocorrelations as a within-species temporal interdependency (intraspecific). We can note how for the largest capitalizations, when the interspecific correlations are relevant and positive, the intraspecific ones are generally much smaller and statistically not significant. In this case, interspecific correlations are not weak in comparison with intraspecific ones and could be generated by some constraints on the community dynamics not generated by neutral dynamics.

To sum up, we have developed an analysis of the cryptocurrency market borrowing methods and concepts from ecology. This approach allows for identifying specific diversity patterns and their variation, in close analogy with ecological systems, and to describe them effectively. At the same time, we can contrast different ecological theories, testing the validity of using neutral models. The behaviour of the SAD and the evolution of the community structure strongly suggest that these statistical patterns are not consistent with neutrality. In particular, the necessity to increase the community composition changes when species richness increases suggests that the interactions among cryptocurrencies are not necessarily weak. This fact is supported by the analysis of intraspecific and interspecific interdependency, which also demonstrates that a market sector influenced by mutualistic relations can be outlined. All these outcomes challenge the hypothesis of weakly interacting symmetric species.

Our results show that the community structure of the cryptocurrency market can be effectively described by using an ecological perspective. Our analysis, besides static distributions, highlights specific patterns of a rich temporal dynamics. These data were compared directly with neutral models. Even if falsified, these models offer a reference point for parsimonious description, acting as a useful null model. Our study introduces interesting new strategies for describing dynamical patterns that are novel even for ecological studies. The accessibility to the whole dataset of the cryptocurrency market, without limitations derived from sampling, makes possible the exploration of these tools. For these reasons, these results have an interesting impact either in the characterization of the cryptocurrency market, or on the relevance that non-biological systems can have in testing ecological theories. Finally, the new set of empirical regularities and general tendencies displayed by the cryptocurrencies market could introduce interesting elements of exploration for the exciting and emerging field of market ecology [36,37].

## Data Availability

All the data used in this article can be downloaded from the website Coin Market Cap. The downloading procedure is accessible at: https://coinmarketcap.com/all/views/all/.

## References

[1] Nakamoto S. 2009 Bitcoin: a peer-to-peer electronic cash system. See https://bitcoin.org/bitcoin.pdf.

[2] Ammous S. 2018 Can cryptocurrencies fulfill the functions of money? Q. Rev. Econ. Finance **70**, 38-51. (10.1016/j.qref.2018.05.010)

[3] Urquhart A. 2016 The inefficiency of Bitcoin. Econ. Lett. **148**, 80-82. (10.1016/j.econlet.2016.09.019)

[4] Dimitrova V, Fernández-Martínez M, Sánchez-Granero MA, Trinidad Segovia JE. 2019 Some comments on Bitcoin market (in)efficiency. PLoS ONE **14**, e0219243. (10.1371/journal.pone.0219243) 31283773 PMC6613746

[5] De Sousa Filho FNM, Silva JN, Bertella MA, Brigatti E. 2021 The leverage effect and other stylized facts displayed by Bitcoin returns. Braz. J. Phys. **51**, 576-586. (10.1007/s13538-020-00846-8)

[6] Drożdż S, Minati L, Oświe¸cimka P, Stanuszek M, Watorek M. 2020 Competition of noise and collectivity in global cryptocurrency trading: route to a self-contained market. Chaos **30**, 023122. (10.1063/1.5139634) 32113224

[7] ElBahrawy A, Alessandretti L, Kandler A, Pastor-Satorras R, Baronchelli A. 2017 Evolutionary dynamics of the cryptocurrency market. R. Soc. Open Sci. **4**, 170623. (10.1098/rsos.170623) 29291057 PMC5717631

[8] Wu K, Wheatley S, Sornette D. 2018 Classification of cryptocurrency coins and tokens by the dynamics of their market capitalizations. R. Soc. Open Sci. **5**, 180381. (10.1098/rsos.180381) 30839686 PMC6170580

[9] Gaston KJ, Blackburn TM, Lawton JH. 1993 Comparing animals and automobiles: a vehicle for understanding body size and abundance relationships in species assemblages? Oikos **66**, 172-179. (10.2307/3545211)

[10] Blonder B, Sloat L, Enquist BJ, McGill B. 2014 Separating macroecological pattern and process: comparing ecological, economic, and geological systems. PLoS ONE **9**, e112850. (10.1371/journal.pone.0112850) 25383874 PMC4226609

[11] Keil P, MacDonald AAM, Ramirez KS, Bennett JM, García-Peña GE, Yguel B, Bourgeois B, Meyer C. 2018 Macroecological and macroevolutionary patterns emerge in the universe of GNU/Linux operating systems. Ecography **41**, 1788-1800. (10.1111/ecog.03424)

[12] Valverde S, Sole RV. 2015 Punctuated equilibrium in the large-scale evolution of programming languages. J. R. Soc. Interface **12**, 20150249. (10.1098/rsif.2015.0249) 25994298 PMC4590513

[13] Buchanan A, Packard NH, Bedau MA. 2011 Measuring the drivers of technological innovation in the patent record. Artif. Life **17**, 109. (10.1162/artl_a_00022) 21370957

[14] Chow SS, Wilke CO, Ofria C, Lenski RE, Adami C. 2004 Adaptive radiation from resource competition in digital organisms. Science **305**, 84. (10.1126/science.1096307) 15232105

[15] Grinnell J. 1917 The niche-relationships of the California thrasher. The Auk **34**, 427-433. (10.2307/4072271)

[16] MacArthur RH. 1957 On the relative abundance of bird species. Proc. Natl Acad. Sci. USA **43**, 293-295. (10.1073/pnas.43.3.293) 16590018 PMC528435

[17] Hubbell SP. 2001 The unified neutral theory of biodiversity and biogeography (MPB-32). Princeton, NJ: Princeton Univ. Press.

[18] Volkov I, Banavar JR, Hubbell SP, Maritan A. 2003 Neutral theory and relative species abundance in ecology. Nature **424**, 1035-1037. (10.1038/nature01883) 12944964

[19] Borile C, Muñoz MA, Azaele S, Banavar JR, Maritan A. 2012 Spontaneously broken neutral symmetry in an ecological system. Phys. Rev. Lett. **109**, 038102. (10.1103/PhysRevLett.109.038102) 22861902

[20] Azaele S, Pigolotti S, Banavar J, Maritan A. 2006 Dynamical evolution of ecosystems. Nature **444**, 926-928. (10.1038/nature05320) 17167485

[21] Fisher CK, Mehta P. 2014 Niche-to-neutral transition in ecology. Proc. Natl Acad. Sci. USA **111**, 13 111-13 116. (10.1073/pnas.1405637111) PMC4246938 25157131

[22] Total Market Capitalization. See https://coinmarketcap.com/all/views/all/.

[23] McGill BJ, Maurer BA, Weiser MD. 2006 Empirical evaluation of neutral theory. Ecology **87**, 1411-1423. (10.1890/0012-9658(2006)87[1411:EEONT]2.0.CO;2) 16869415

[24] Fisher RA, Corbet AS, Williams CB. 1943 The relation between the number of species and the number of individuals in a random sample of an animal population. J. Anim. Ecol. **12**, 42-58. (10.2307/1411)

[25] Preston FW. 1948 The commonness, and rarity, of species. Ecology **29**, 254-283. (10.2307/1930989)

[26] McGill BJ. 2003 A test of the unified neutral theory of biodiversity. Nature **422**, 881-885. (10.1038/nature01583) 12692564

[27] May RM.1975 Patterns of species abundance and diversity. In Ecology and Evolution of Communities (eds ML Cody, JM Diamond), pp. 81-120. Belknap, Cambridge: Harvard Univ. Press.

[28] Connolly SR et al. 2014 Commonness and rarity in the marine biosphere. Proc. Natl Acad. Sci. USA **111**, 8524-8529. (10.1073/pnas.1406664111) 24912168 PMC4060690

[29] Azaele S, Suweis S, Grilli J, Volkov I, Banavar JR, Maritan A. 2016 Statistical mechanics of ecological systems: neutral theory and beyond. Rev. Mod. Phys. **88**, 035003. (10.1103/RevModPhys.88.035003)

[30] Dornelas M, Gotelli NJ, McGill B, Shimadzu H, Moyes F, Sievers C, Magurran AE. 2014 Assemblage time series reveal biodiversity change but not systematic loss. Science **344**, 296-299. (10.1126/science.1248484) 24744374

[31] Magurran AE, Dornelas M, Moyes F, Henderson PA. 2019 Temporal β diversity – a macroecological perspective. Global Ecol. Biogeogr. **28**, 1949-1960. (10.1111/geb.13026)

[32] McGill BJ, Hadly EA, Maurer BA. 2005 Community inertia of quaternary small mammal assemblages in North America. Proc. Natl Acad. Sci. USA **102**, 16 701-16 706. (10.1073/pnas.0504225102) PMC1283796 16260748

[33] Engen S, Lande R, Walla T, DeVries PJ. 2002 Analyzing spatial structure of communities using the two-dimensional poisson lognormal species abundance model. Am. Nat. **160**, 60-73. (10.1086/340612) 18707499

[34] Pigolotti S, Flammini A, Maritan A. 2004 Stochastic model for the species abundance problem in an ecological community. Phys. Rev. E **70**, 011916. (10.1103/PhysRevE.70.011916) 15324097

[35] Gaston KJ. 2011 Common ecology. BioScience **61**, 354. (10.1525/bio.2011.61.5.4)

[36] Farmer JD. 2002 Market force, ecology and evolution. Ind. Corp. Change **11**, 895-953. (10.1093/icc/11.5.895)

[37] Scholl MP, Calinescu A, Farmer JD. 2021 How market ecology explains market malfunction. Proc. Natl Acad. Sci. USA **118**, e2015574118. (10.1073/pnas.2015574118) 34172576 PMC8256038

